# User Retention and Engagement With a Mobile App Intervention to Support Self-Management in Australians With Type 1 or Type 2 Diabetes (My Care Hub): Mixed Methods Study

**DOI:** 10.2196/17802

**Published:** 2020-06-11

**Authors:** Mary D Adu, Usman H Malabu, Aduli EO Malau-Aduli, Aaron Drovandi, Bunmi S Malau-Aduli

**Affiliations:** 1 College of Medicine and Dentistry James Cook University Townsville Australia; 2 College of Public Health, Medical and Veterinary Sciences James Cook University Townsville Australia

**Keywords:** mobile apps, engagement, retention, diabetes mellitus, self-management, behavioral intervention technology

## Abstract

**Background:**

Mobile health apps are commonly used to support diabetes self-management (DSM). However, there is limited research assessing whether such apps are able to meet the basic requirements of retaining and engaging users.

**Objective:**

This study aimed to evaluate participants’ retention and engagement with My Care Hub, a mobile app for DSM.

**Methods:**

The study employed an explanatory mixed methods design. Participants were people with type 1 or type 2 diabetes who used the health app intervention for 3 weeks. Retention was measured by completion of the postintervention survey. Engagement was measured using system log indices and interviews. Retention and system log indices were presented using descriptive statistics. Transcripts were analyzed using content analysis to develop themes interpreted according to the behavioral intervention technology theory.

**Results:**

Of the 50 individuals enrolled, 42 (84%) adhered to the study protocol. System usage data showed multiple and frequent interactions with the app by most of the enrolled participants (42/50, 84%). Two-thirds of participants who inputted data during the first week returned to use the app after week 1 (36/42, 85%) and week 2 (30/42, 71%) of installation. Most daily used features were tracking of blood glucose (BG; 28/42, 68%) and accessing educational information (6/42, 13%). The interview results revealed the app’s potential as a behavior change intervention tool, particularly because it eased participants’ self-care efforts and improved their engagement with DSM activities such as BG monitoring, physical exercise, and healthy eating. Participants suggested additional functionalities such as extended access to historical analytic data, automated data transmission from the BG meter, and periodic update of meals and corresponding nutrients to further enhance engagement with the app.

**Conclusions:**

The findings of this short-term intervention study suggested acceptable levels of participant retention and engagement with My Care Hub, indicating that it may be a promising tool for extending DSM support and education beyond the confines of a physical clinic.

## Introduction

### Background

Mobile health (mHealth) apps offer a unique opportunity to deliver health promotion interventions to reach any population due to their ubiquitous nature [1,2], with some developed specifically to support diabetes management [3,4]. However, these mHealth interventions suffer from low participant retention [5,6] and nonusage attrition [6,7]. Therefore, more engaging interventions are required to address these concerns [8,9] through user-centered and iterative approaches that integrate input from users and other relevant stakeholders in app design and development. This approach is necessary to provide interventions that meet user requirements and ensure greater retention, uptake, engagement, and sustainability [9,10].

### Retention

Inadequate participant retention is a major methodological challenge experienced by many mHealth app interventions [11]. Low retention rates and lower statistical power threaten outcome validity [6] and serve as a major reason for premature trial termination [12]; hence, pilot studies are important before conducting large-scale studies. The evaluation of participant retention levels enables researchers to assess the relevancy and tendency for sustainable implementation of intervention ideas [13]. In addition, the assessment reveals any required research methodology modification [13] in preparation for future large-scale research.

### Engagement

An effective mHealth intervention requires not only retention but also continuous and active engagement by users, as lack of engagement leads to study dropout and dampening of the treatment effect [6,11]. User engagement refers to interaction, experience, perceived usefulness, and desire to use the intervention repeatedly over a long period of time [14,15]. The degree to which users engage with a health app signifies their willingness to invest time, attention, and emotion into the use of the technology to satisfy and eventually achieve their pragmatic needs (such as self-management) [14]. Measurement of users’ engagement can be long or short term in nature with short-term measurement reflecting initial adoption of the intervention and the tendency of apps to successfully engage users in the long term [14]. Although, there are various approaches to measuring engagement with apps, system usage data and user-reported interactions with the system using specific techniques such as questionnaires and interviews are the most relevant in the context of short-term measurement [9,14,16].

System usage is measured through the collection of noninvasive data on the frequency of access to the app, push notifications opened, and average time spent per usage [17,18]. This provides information on user participation with specific target behaviors and frequency of access to the corresponding app features [19,20]. On the other hand, user-reported approaches reveal users’ experiences related to behavioral engagement with the intervention [14,16]. This is necessary to assess intervention tendency to foster achievement of behavioral goals when used over a long period.

### Behavioral Engagement Framework

Rate of use alone is not a sufficient indicator of engagement with an mHealth intervention [9]. There must also be an assessment of engagement with the behavioral goal of the intervention to ascertain the intervention’s potential as an effective tool to support behavioral change. One possible way to achieve this is by assessing users’ engagement with the process of achieving behavioral change. Behavioral change is fostered by intervention components that motivate users to achieve a behavioral goal (in this case, diabetes self-management (DSM) behaviors) [9]. Assessing engagement in behavioral change process requires the use of models and frameworks that reveal the relationship between factors in a system for the realization of a defined goal [21].

Within the field of mHealth engagement, models and frameworks provide a richer understanding of the core components that influence user engagement to achieve the behavioral goal of the intervention [22]. The concept of behavioral engagement is complex and includes the extent to which users interact with the intervention. Major considerations include the quality of users’ experience with the technology [23] and if they have engaged with it as needed [7] or as intended [23]. The behavioral intervention technology (BIT) model by Mohr et al [24] describes the full range of components that must be available in a technology to influence engagement with behavioral change and its potential as an effective intervention to attain a behavioral goal.

The BIT framework [24] was utilized in this study as it describes the theoretical components necessary in the conceptualization of mHealth and also instantiates the necessary components for its implementation. The theoretical level covers the overall goal (*why*) or reason for mHealth development and *how* specific aims related to the goal could be achieved through the required *behavioral change strategies*. Each strategy is instantiated by *elements*: features (*what*) available in the intervention. In addition, the *characteristics (technic)* of the intervention affect *how* an element is displayed to the users as well as their perception about the intervention. Finally, the *workflow (pattern of use)* describes *when* and under what conditions BIT interventions will be delivered. Therefore, the BIT model explains that achieving an intervention goal is fostered through relationships between the components *of aims, behavioral change strategies, elements, characteristics, and pattern of use* of the intervention [24]. We used this model to interpret our qualitative findings, allowing for an open approach to the concept of behavioral engagement, focusing on exploring the tendency of My Care Hub as an intervention tool for diabetes behavioral engagement.

### Study Context and Objectives

Owing to poor retention and engagement with previous diabetes apps, we performed an initial study to explore user needs and preferences to foster engagement with a diabetes app [25], which was used to develop a new app called My Care Hub [26]. Patients with diabetes who interacted with a prototype of My Care Hub reported that it was easy to use and that the educational contents were valuable in raising awareness about the importance of DSM and increased motivation to engage in self-management activities [26]. Although the usability of the app was satisfactory, it was unclear if My Care Hub has the potential to retain and engage users and if its components meet the requirements of a supporting tool to foster engagement with DSM.

Therefore, this study aimed to examine levels of user retention and engagement with My Care Hub in a short-term single-arm pilot trial. Retention was measured through completion of follow-up surveys, and engagement with the app was assessed in 2 areas: (1) system usage data and (2) qualitative feedback from users on behavioral interactions with the intervention. We expect that the app’s contents and features, which were developed based on results from our previous study on users’ needs [25,26], would result in high participant retention and greater engagement during the short trial period. Understanding these factors is critical in identifying areas where intervention design may need improvement and inform plans for future trials of mHealth interventions such as My Care Hub.

## Methods

This study received ethics approval from the James Cook University Human Research Ethics Committee (reference #H7716). Participants were informed about the study aims, and consent was implied by survey submission. Verbal consent was obtained for telephone interviews.

### Study Design and Sample Size

This study utilized a sequential explanatory mixed methods design with quantitative surveys and qualitative interviews. This design captures both the engagement with technology and the process of behavioral change by triangulating the results of multiple measures [27]. This provides information about how users react to the contents and design of the intervention and offers an explanation for why users interact with the intervention in a particular way [9]. This study was conducted from August to October 2019, where each participant was given 3-week access to the app. Following this period, participants filled out a survey and were invited to participate in a telephone interview to better understand their interaction with the app.

The study used a maximum variation purposive sampling tailored to recruit participants who showed interest in the study within the time available. This sampling method is appropriate for an implementation feasibility assessment as related to this study [28]. The components of the pilot testing that relate to retention and engagement with the app are presented in this paper.

### Recruitment and Eligibility

Participants were recruited through a single invitation email sent to patients registered with the Australian National Diabetes Service Scheme. Email invitations were limited to patients who have type 1 or type 2 diabetes and live in North Queensland, Australia. North Queensland has a relatively high prevalence of diabetes [29] and socioeconomic disadvantage, which can affect accessibility to regular diabetes support services [30]. Therefore, the use of mHealth interventions to provide DSM support may be essential among this population. Other eligibility criteria included ownership of an Android-operating smartphone, having a current recommended blood glucose level (BGL) target of 4 to 10 mmol/L [31], and being aged 18 to 65 years. The upper age limit was chosen because of the less stringent glycemic recommendations for many older adults who are above 65 years. Patients were excluded if pregnant or currently using an app with an educational component to support their diabetes management.

### Enrollment and App Orientation

Participants enrolled through the web by completing an eligibility screening form, providing consent, and completing the baseline survey, which entailed questions regarding socio-health demographics, email address, and residential postcode. Participants were emailed a unique code to enable them to download My Care Hub from Google Play store of any android-powered phone, an app manual, and a 5-min video explaining how to install the app, features, and functionalities. Participants could contact the first-named author (MA) for assistance with technical difficulties or for study clarification. It was emphasized that there was no limit to the frequency of use of My Care Hub as participants could engage with it at a level they considered useful and desired. My Care Hub is intended to be a stand-alone intervention; therefore, push notifications (aimed at improving patients’ awareness about diabetes distress and potential ways to reduce its impact on their self-management) were sent from the app during the first 2 weeks of the intervention and withheld in the third week to see the achievable level of engagement with the app with or without push notifications. Throughout the study period, no log-in reminders or calls were made from the study researchers to participants.

### Intervention Overview

A detailed description of the development of My Care Hub and the methods of usability studies have been previously published [26]. In brief, the goal of My Care Hub is to provide support and education that facilitates positive behavioral change in diabetes management. The app was specifically designed for type 1 diabetes patients with standard Australian BGL recommendations of 4 to 8 mmol/L for fasting and <10 mmol/L 2-hour postprandial, and for type 2 diabetes patients with recommended fasting BGL of 6 to 8 mmol/L and 2-hour postprandial levels of 6 to 10 mmol/L. The app incorporates multiple functions and features to foster engagement with the app within 3 broad categories: *documentation*, *analytics*, and *education*.

In *documentation features*, users can manually input data for tracking BGL, physical activity, the carbohydrate content of foods eaten, and body weight. *Analytic features* provided a graphical output of each documentation feature, thus offering users the ability to visually inspect their logged data over time. *Education* was provided through 4 main features. First, users can review a variety of actionable textual information related to healthy food choices, self-monitoring of BGL, medication, reducing risk, healthy coping, problem solving, and physical activities. Second, users can look up information related to carbohydrate and calorie content of common foods in Australia (categorized under fruits and vegetables, egg and meat, dairy, grain and legumes). Third, the BGL feature provided immediate tailored feedback to every inputted data, driven by a decision-based system. The system is controlled by the value of logged BGL (either within or beyond the standard range), type of diabetes, and the indicated period of BGL measurement (either fasting or 2 hours postprandial). Messages were health-promoting and motivational information aimed at supporting behavioral skills building for self-management practices. Finally, the app provided education through daily push notifications aimed at improving awareness about diabetes distress and encouraging patients to focus on potential ways to reduce its impact on their self-management. Push notifications were terminated at the end of the second week. Sample screenshots are provided in Multimedia Appendix 1.

### Postintervention Data Collection

At the end of the study, participants were sent an email (with 1 reminder email sent to noncompleters), which directed them to the poststudy survey on the acceptability of the app and its preliminary efficacy (*results will be reported in future publications*). Through this survey, participants were also invited to participate in individual telephone interviews to further understand their perception of the app. Participants who completed the poststudy survey were awarded an electronic gift (e-gift) card worth Aus $40 (US $25.07). All telephone interviewees were contacted within 3 weeks of completing the survey and awarded an additional Aus $20 (US $12.53) e-gift card.

### Measures

Retention was assessed using the following indicators of study completion per protocol: number of participants enrolled, number of participants who used the app during the intervention period, and completion rate of the poststudy survey.

Engagement with My Care Hub was measured using participants’ app usage log and verbal feedback. App usage data were extracted from the app’s activity database. The following time frames were considered: (1) date of log-in into the app to 2 weeks of use when the daily push notification was administered (referred to as week 1 and week 2) and (2) data during the third week (referred to as week 3) after the termination of push notifications. Key metrics collected from the database included app use (number of active users, frequency of daily access to app), data logs/time spent (for BGL, exercise, food activity, and weight), and number of opened notifications. Metrics were presented using an adapted version of the Frequency, Intensity, Time, and Type (FITT) principle index [32,33]. This index explores multidimensional domains of usage data, which provides greater insight into interaction with an mHealth app. Event count in the app was available for active (documentation features) and passive (viewing of educational screen) app features. Data had to be logged/saved in the documentation features before it could be counted as an active event as the app discarded data not logged after 30 min of inactivity. Users had to exit from an educational screen before it was counted as a passive event, and no maximum count per user was stipulated. This implies that the total count of passive events could be higher if a screen was viewed more than once. The FITT index used in this study is as follows:

1. Frequency index (*F_i_*): This subindex is an attention proxy that provides information on how often a participant uses the app. It recognizes the number of users who return to use the app and active app users in each time period.
2. Intensity index (*I_i_*): This subindex denotes the proportion of users who interact with each feature in the app. In total, 2 metrics were used in the assessment of *I_i_*. These are the frequency of daily use of app features (*I_i1_*) and number of push notifications opened versus the total sent (n=14) in 2 weeks (*I_i2_*). In addition, intensity also measures the proportion of app features used out of the total available features.
3. Type index (*T_y_*): This provides information on the form of engagement based on actions performed by users using the available app features. In this study, the type of action was categorized as *active* denoted as *T_ya_* (use of documentation features for self-monitoring), and *passive* (*T_yp_*), reading information on educational contents in the app).
4. Time index (*T_i_*): This measures the duration of engagement, which signifies attention to the app as a function of daily event duration with each app feature.

### Interviews

Interviews were conducted using a semistructured interview guide that explored behavioral engagement with the app through questions on patterns of use, perceived ease of use, perceived usefulness of app features enabling motivation for continued engagement with DSM, and recommendations on how the app could be improved. The interview guide has been provided in Multimedia Appendix 2.

Interviews were conducted by 1 author (AD), who is well experienced in qualitative research. The interview guide was pilot tested between MA and AD before actual use. The interviewer was located in a private office at James Cook University, Australia, while participants were asked if they were in a comfortable location before commencement of the interview. The first 3 interviews were used to reflect on the guide, although there were no resultant changes. Data saturation was achieved as judged by no emerging new information [34] after completing the 15th (of 17) interview. Interviews were audio recorded, and none of the participants had a previous relationship with any of the authors.

### Data Analysis

Descriptive statistics were calculated for all quantitative variables. Baseline characteristics comparison between those who completed the study and those who did not were done using a Pearson chi-square test. All statistical analyses were performed using SPSS version 23 [35].

Interviews were completed in an average of 15 min (range 9-30 min). Participant responses were transcribed verbatim by 1 researcher (AD). In this analysis, a combination of data and a concept-driven strategy was applied. Initially, inspired by the work of Schreier [36], 2 researchers (MA and AD) independently used a data-driven strategy to obtain an overview of the data, and then similar text segments were selected and sorted using coding. Coded segments were grouped to identify recurring themes from the data. Themes were compared between the 2 authors, discussed with a third author (BM), adjusted, and an agreement was reached about the main themes. Subsequently, the authors analyzed the themes by applying a concept-driven strategy in accordance with the BIT framework [24] to assess behavioral engagement with the intervention. We identified and described the BIT components in the My Care Hub app that could potentially enhance behavioral engagement with it. These components overlap and diverge within the identified themes, which are presented using representative quotes affixed with an assigned number code and the type of diabetes the respondent has (for instance, respondent 3 with type 1 diabetes; P003, T1D and respondent 4 with type 2 diabetes; P004, T2D). The conduct and reporting of the interviews followed the consolidated criteria for reporting qualitative research (Multimedia Appendix 3) [37].

## Results

### Participant Characteristics

Participant demographics and health characteristics are shown in Table 1. Participants were predominantly male (31/50, 62%), had type 2 diabetes (36/50, 76%), and aged between 20 and 64 years (mean 49.12, SD 12.34 years). On average, the recommended BGL in enrollees was as follows: for fasting, 4.58 (SD 0.78; range 4-6 mmol/L), and for 2-hour postprandial, 7.01 (SD 1.02; range 6-10 mmol/L). Most participants were diagnosed as having diabetes in the last 5 years (27/50, 54%), and an equal proportion rated their health status as being fair or good (20/50, 40%). Most had a technical college education or higher (39/50, 78%) and were employed (31/50, 62%). Only a few had previously used a health app to manage diabetes in the past (16/50, 32%). The linking of participants’ postcode to the Australian Standard Geographical Classification System [38] indicates the geographic location of the majority to be rural (37/50, 74%).

Of the 22 participants who indicated an interest in participating in the interview, only 17 were contactable within 3 call attempts. Most were males (12/17, 71%), had type 2 diabetes (13/17, 77%), and had been diagnosed for an average of 6 years (range 1-17 years). Overall, participants were between the ages of 36 to 64 years (mean 51.58, SD 11.31), except for one who was aged 20 years.

### Retention

Of the 4984 patients who were emailed an invitation to participate in the study, 79 (1.59%) completed the eligibility form. However, only 84% (67/79) of those who responded met the inclusion criteria and were provided access to download the app. Some participants (17/67, 25% of those eligible) failed to log in to the app, resulting in 50 enrolled participants (75% of eligible participants). Most enrollees (43/50, 86%) activated the app within the same day (range 0-5 days) of having access to it. One participant logged out of the app on the second day of installation stating that it did not meet her requirement. At the end of the study period, 41 of the enrolled participants completed the study per protocol by providing feedback about the app using the poststudy survey (retention rate: 41/50, 82%). Reasons for noncompletion of the study protocol were not recorded. In assessing baseline characteristics associated with retention, only employment status emerged as a significant predictor, with those unemployed being less likely to complete the study than those who were employed (50.0% versus 14.7%, respectively; *P*=.02). The full details of the demographic variables and comparison between those who completed the study and those who did not are shown in Table 1.

**Table 1:** Participant characteristics.

Characteristics		Baseline (N=50)	Completers (n=41), n (%)	Lost to follow-up (n=9), n (%)		*P* value
**Gender**					.75	
	Male	31	25 (81)	6 (19)		
	Female	19	16 (84)	3 (16)		
Age (years), mean (SD)		N/A^a^	49.29 (12.74)	48.67 (11.25)		.82
**Age (years)**					.82	
	18-29	5	4 (80)	1 (20)		
	30-39	6	5 (83)	1 (17)		
	40-49	12	10 (83)	2 (17)		
	50-59	15	11 (73)	4 (27)		
	60-65	12	11 (92)	1 (8)		
**Type of diabetes, n (%)**					.81	
	Type 1	15	12 (80)	3 (20)		
	Type 2	35	29 (83)	6 (17)		
**Type 2 medications or not, n (%)^b^**					.32	
	None	2	1 (50)	1 (50)		
	Oral drugs alone	33	28 (85)	5 (15)		
	Oral and insulin	1	1 (100)	0 (0)		
**Duration of diagnosis (years), n (%)**					.92	
	<5	27	23 (85)	4 (15)		
	6-10	10	8 (80)	2 (20)		
	11-15	9	6 (67)	3 (33)		
	>16	4	4 (100)	0 (0)		
**Education, n (%)**					.59	
	High school equivalent	17	12 (71)	5 (29)		
	Technical college	10	9 (90)	1 (10)		
	First degree	11	10 (91)	1 (9)		
	Postgraduate	8	7 (88)	1 (12)		
	Missing	4	3 (75)	1 (25)		
**Ethnicity, n (%)**					.87	
	Caucasian/white	47	38 (81)	9 (19)		
	Missing	3	3 (100)	0 (0)		
**Employment, n (%)**					.02^c^	
	Unemployed	8	4 (50)	4 (50)		
	Partly/fully employed	34	29 (85)	5 (15)		
	Retired	8	8 (100)	0 (0.00)		
**Living environment, n (%)**					.26	
	Remote	13	12 (92)	1 (8)		
	Rural	37	29 (78)	8 (22)		
**Usage of smartphone (years), n (%)**					.42	
	1-5	13	11 (85)	2 (15)		
	6-10	28	24 (86)	4 (14)		
	>10	9	6 (67)	3 (33)		
**Previous use of health apps to manage diabetes, n (%)**					.93	
	Yes	16	13 (81)	3 (19)		
	Never	34	28 (82)	6 (18)		
**Rating of health status, n (%)**					.38	
	Poor	1	1 (100)	0 (0)		
	Fair	19	14 (74)	5 (26)		
	Good	21	17 (81)	4 (19)		
	Very good	9	9 (100)	0 (0)		

^a^N/A: not applicable.

^b^N=35.

^c^*P*<.05.

### App Engagement

Most (42/50, 84%) enrolled participants logged data into the app at least once (during week 1 of installation) with the frequency index showing that they actively used the app on an average of 11 of the 14 days in the first 2 weeks when push notifications were sent (range 2-14 days; week 1 average: 5.2 days, week 2 average: 4.8 days). This reduced to an average of 4 of 7 days (range 2-5) in week 3: average 3.8 days. Furthermore, all participants who logged in to the app used it during week 1, and most returned to use the app after week 1 (36/42, 85%) and week 2 (30/42, 71%) of installation. With regard to the intensity index related to daily use of each app feature (*I_i1_*), most participants used features for tracking their BGL (28/42, 68%) and accessing educational information (6/42, 13%) more frequently. The feature with the least daily use was tracking the carbohydrate content of foods (2/42, 2%). All 14 push notification messages during the first 2 weeks (1 per day) sent were published, and on average, 57% (24/42) of participants opened this notification within 24 hours, after which they were automatically deleted. None of the app features were unused. The type index (*T_y_*) shows active and passive actions with the My Care Hub. The average frequency of BGL data log per participant in week 1 was 10.85 (SD 9.32; range 1-36), which reduced to 6.75 (SD 7.75; range 1-24) in week 2 and 5.67 (SD 6.05; range 0-22) in week 3. Physical activity logs showed a mean of 4.48 (SD 3.64; range 0-15) in week 1 compared with 2.97 (SD 2.93; range 0-11) in week 2 and 1.69 (SD 1.70; range 0-7) in week 3. Average passive engagement per participant on occasions of viewing screens alone in week 1 was 26.5 (SD 2.51; range: 9-32), 17.55 (SD 7.39; range 7-26) in week 2, and 14.4 (SD 6.13; range 6-24) in week 3. The time index (*T_i_*) revealed that, for all events of participants’ visit to the app, an average daily time of 3.56 min (range 1.37-7.48 min) was spent. More time was spent on BGL activity (2.2 min) and accessing the educational tips embedded in the app (1.35 min). Table 2 summarizes the app functions and features, their purposes, usage, and engagement.

**Table 2:** My Care Hub sections and engagement indices (N=42).

Functions and features		Elements		Purpose		User engagement			
						Percentage of daily users (*I_i1_*)^a^, n (%)		Average time spent per user per day (*T_i_*)^b^	
**Documentation**									
	BG^c^ activity (*T_ya_*)^d^		•BG log •Type of BG •Automatic feedback (as part of education)		•Monitoring and tracking of BG values over time •Gain knowledge to support self-management practices		29 (69)		2 min 2 seconds
	Physical activity (*T_ya_*)		•Log of time spent on physical activity •Calories used •Place		•Monitoring of physical activity behavior over time		4 (10)		0 min 7 seconds
	Food activity (*T_ya_*)		•Record of food intake •Log of carbohydrate content of food		•Monitoring and tracking of food intake and their carbohydrate content over time		1 (2)		0 min 17 seconds
	Weight log (*T_ya_*)		•Body weight log		•Body weight assessment over time		2 (5)		0 min 22 seconds
	Analytics (*T_yb_*)^e^		•Graphical display of data log into each documentation feature		•Keeping track of trends in lifestyle activities and observe impact on BGL^f^ over time		3 (6)		0 min 20 seconds
**Education**									
	Textual screens for management tips and food choices (*T_yb_*)		•Information on behaviors in DM^g^ management •Information on average carb and calorie content of common Australian foods		•Assess current knowledge on DSM^h^ •Review carbohydrate content of foods to make healthy choices.		6 (13)		1 min 35 seconds
	Push notifications (*T_yb_*) and (*I_i2_*)^i^		•Messages on diabetes distress		•Create awareness about diabetes distress and ways to reduce its impact on self-management		24 (57)		^—j^

^a^*I_il_*: intensity index for frequency of daily use.

^b^*T_i_*: time index.

^c^BG: blood glucose.

^d^*T_ya_*: type index for active app use.

^e^*T_yb_*: type index for passive app use.

^f^BGL: blood glucose level.

^g^DM: diabetes management

^h^DSM: diabetes self-management.

^i^*I_i2_*:intensity index for number of push notifications opened.

^j^Not captured due to the tracking limitations of the system usage database.

### Interview Results

Different themes emerged from the data with interconnection among the themes over the course of My Care Hub usage. Overall, the results suggest that the use of the app has the potential to ease the effort in aiming for improved self-management and for better awareness of BGLs. In addition, participants provided their recommendations for extra functionalities that may further enhance engagement with self-management behaviors. We present our findings in relation to themes related to components of the behavioral intervention model [24] used for this study, which are outlined in Table 3.

**Table 3:** Summary of behavioral intervention technology model as adapted to My Care Hub intervention.

BIT^a^ components			BIT^a^ components		Details in MCH^b^
**Theoretical**					
	Why	Broader goal: self-management support		Aims: Improved BG^c^—long-term impact Increased physical activity Healthy eating Decreased diabetes stress	
	How	Behavioral change strategies		Elements or strategies	
				Documentation and Analytics: Accountability; Clarity of self-management activities and impact; Improved awareness of BG^c^ levels; Mindfulness of calorie consumption Feedback response: Reinforced recommendation of HP^d^; Informative Carbohydrates in foods: Guidance on meal planning; Knowledge provision and reinforcement Educational tips: Knowledge reinforcement	
**Instantiation**					
	What	Elements (app features)		Documentation (logs)and analytics: Feedback response Carbohydrates in foods Educational tips screen Push notifications	
	How (technic)	Characteristics		Aesthetic: Beautiful Ease of use: Simple and straight forward Few difficulties	
	When	Pattern of use		User defined Type of diabetes Established self-management routines Frequency: Daily Partly, with reasons	

^a^BIT: behavioral intervention technology.

^b^MCH: My Care Hub.

^c^BG: blood glucose.

^d^HP: health provider.

### Pattern of Use (When)

#### User Defined

Patterns of app use depended on users’ type of diabetes and self-management routines, with most participants using the app multiple times per day, where those with type 1 diabetes input their BGL any time it was measured:

I use it multiple times per day, basically any BGL I took I enter it at any time I took it.

P001,T1D

In contrast, participants with type 2 diabetes described that the frequency of usage depends on the self-management activity carried out on that day.

I used it at least once a day. if I had done exercise, then I was putting in an exercise and blood test virtually every day. On every second day I was using it to stick in weight but the exercise was done at a different time.

P005,T2D

Conversely, some participants were only able to use the app infrequently because of issues such as limited internet access or multiple competing interests:

I didn’t use it fully, because at the moment I am having a problem with my internet, so I didn’t get a chance to watch the video that comes with it.

P012,T2D

I used it a few times to start with, but then I stopped pretty much because I was juggling between doing a lot of writing, doing a course, and was having other things to do.

P003,T1D

### Characteristics of the App (How)

#### Simple and Straightforward

Participants described the design as:

Very well crafted and well put together, really easy to use [P007, T2D],

and could be used even by the elderly who may not be too proficient in using mobile technology:

I would even say that like an older person in their 60s or so, once they get an idea of how to use it properly, would have no worries using it if they were in that way inclined.

P012,T2D

#### App Difficulties

Some participants found a few aspects of the app difficult:

There was one for the activities you had to put in what calories you might have burned off and I didn’t have a clue how I was going to find out that information.

P013,T2D

I had a problem figuring out how to put dates in it, but I think it does it itself, so yeah.

P008,T2D

### Goal (Why), Elements (App Features; What), and Behavioral Change Strategies (How)

The goal of developing My Care Hub was to enhance engagement with self-management activities such as improved BG, increased participation in physical exercise, and healthy eating. Participants identified multiple elements (features) that support this overall goal. They also described the perceived benefits (mechanism of action) of each of the elements that encouraged their interaction with it, and toward achieving an improved DSM. The commonly mentioned features are noted below, as well as reasons why participants found the features engaging.

#### Documentation/Analytics

##### Accountability

Participants mentioned that the documentation element strengthened the sense of responsibility to keep up with routines in DSM:

I liked the activity log, because it gives you accountability, when did you go to the gym, how long were you there, what did you do.

P014,T2D

##### Clarity of Self-Management Activities and Impact

Participants explained that visualization of logged data using *analytics* encouraged their interaction with My Care Hub. They noted that the feature provides better clarity on their level of self-care:

Just the tracking of my fitness, exercise and my blood sugars, it is much better for me seeing it in a graph, makes it really clear how you are going.

P006,T2D

The feature also hinted at some participants to consult their physician for medication review or consultation if their BGLs were not in the recommended range:

I liked the graphs…, that was what gave me the red flag…maybe I have to see the doctor to have my medications changed.

P010,T2D

##### Improved Awareness of Blood Glucose Levels

Participants noted that although they have a BG meter that provides BG measurement history, having the graphical output of their BGL in My Care Hub further improved awareness of any fluctuations in BGLs:

It was quite good to see longitudinal things, obviously on my blood monitor I can see by just hitting the back key what the previous readings are..., But to see it in a graphical linear form was really good. It showed me where my blood sugar was, if I went up and down.

P005,T2D

##### Mindfulness of Calorie Consumption

The analytic feature enabled participants to pay attention to daily calorie intake or carbohydrates consumed:

I liked that idea of putting it all in and seeing how your graphs went up and down, and it sort of kept you a bit more mindful of how many calories or carbs you are eating during the day.

P013,T2D

#### Feedback Response

##### Reinforced Health Provider’s Recommendation

Feedback received in response to logged BGL is an element that reinforced the doctor’s recommendation about participants’ BGLs. A participant with hypoglycemia unawareness noted that his doctor suggested continuing using the app to serve as an alert in the event of low BGL:

It is one thing that made me maintain my BGLs. I tend to be what my doctor calls hypoglycaemia insensitive. So, he suggested that I stick with the app because it reminds me to do regular BGL tests to make sure that I am not dropping too low.

P007,T2D

##### Informative

Feedback feature serves as an alert about a potential problem in users’ BGLs:

I got confirmation that was somewhat reassuring. I mean if it was out and higher, it just alerts you to a potential problem that you may or may not be aware of.

P005,T2D

It aided decision making for improved self-management:

If my levels were over the target range, it gave me very helpful ways to reduce the blood glucose level back into the range.

P007,T2D

#### Carbohydrate Components in Foods

##### Guidance on Meal Planning

Participants valued the *carbs in foods* feature as it provided information about the average carbohydrate and calorie contents of foods. Participants perceived they were better supported in their choice of appropriate foods to eat and avoid exceeding their recommended daily amount of carbohydrate intake. It also provided guidance on food planning:

I try to stay between 20 and 50 grams a day, so the carb counting feature was very useful because then you can make an informed decision on what you are going to put on your plate, and you can plan out your week.

P009,T2D

##### Knowledge Provision and Reinforcement

Participants who had difficulties knowing the carbohydrate content of foods found this feature useful through outlining the best foods for consumption to ensure proper health management:

I have a lot of trouble with how much carbohydrate is in one food but it (app) sort of gets you to realise okay then I have got to check on that.

P004,T2D

Furthermore, engaging with the *carbs in foods* feature reinforced knowledge and served as a reminder about carbohydrate content in foods:

There is so much to take in, like reading labels, it is so much to take in. So I found it (app) quite interesting that it is a bit more set out with carbs and how much is in it, and some of them are low and you thought it would be high. Just reinforcing the information because I just can’t remember everything.

P014,T2D

#### Educational Tips

##### Knowledge Reinforcement

Educational tips were also acknowledged as a tool for knowledge reinforcement and fostered the use of the app. Participants found information on 7 essential ways to manage diabetes quite useful and reflective:

It is useful, I have got a couple of books, and there is a lot of information, and whilst I may have read it, I am not sure I can regurgitate it.

P005,T2D

It was just interesting to read it and think about it.

P014,T2D

In addition, participants felt that the element provided more comprehensive information in comparison with the feedback element:

That (educational tips) was more useful than the little hint things (feedback messages) yeah… I think it probably covered it (all information) fairly thoroughly.

P006,T2D

### Recommendations to Further Improve Engagement With the App

Participants’ recommendations were primarily based on extended functionality in the app, including the following:

1. Automation of data input: Some participants found the manual recording of BGL, physical activity, and carbohydrate content of foods consumed as burdensome and expressed that the addition of Bluetooth, which could automatically extract data from the BGL meter, would not only encourage users’ engagement with My Care Hub but also improve BGL monitoring. Furthermore, the desire for the app to automate the tracking of time spent on physical activities and equivalent calorie expended was expressed. In addition, it was recommended that the app should have features to calculate the calorie content of composite dishes.
2. More analytic histories: Participants suggested extended historical data access and believed this would provide further opportunity to study patterns in self-management activities and have long-term data that could be reviewed by their health care providers.
3. Information update: It was suggested that the Carbs in Foods feature needed more food lists and varieties of composite dishes. Participants suggested that this information could be provided in monthly updates because users’ awareness of finding new information in the app on a regular basis could foster fresh interest in using the app.
4. Feedback on physical activities: The idea of providing motivational feedback in the app, especially when users achieve certain levels of physical exercise, was raised. This behavioral change strategy in My Care Hub is presently limited to the BGL documentation; presumably, participants want an extension of it to the physical activity documentation.

## Discussion

### Overview

The My Care Hub mobile app intervention was intended to encourage ongoing participation in DSM activities. This paper reports the levels of participant retention and engagement (usage and behavioral aspects) with the technology over a 3-week pilot study. The findings of the study revealed an acceptable level of participant retention with the intervention, where the majority completed the study per protocol. Furthermore, participants reported that the intervention eased and improved their effort in participating in self-management activities. Thus, suggesting the app’s potential as a tool for DSM support and education. Nevertheless, a larger sample and longer-term studies are required to establish these claims.

### Participant Retention

The retention rate was relatively high, with more than three-quarters (82%) of participants completing the study per protocol, which is similar to previous short-term pilot studies of diabetes app interventions [39,40]. This indicates that participants were highly motivated and willing to participate in their self-management activities. However, some other pilot studies on DSM support programs reported higher retention than this study. For example, Dick et al [41] reported 0% attrition over 4 weeks, whereas Kim et al [42] reported only 3% loss to follow-up over a 3-month pilot testing. Such findings are expected because the studies [41,42] were conducted in controlled settings where participants’ recruitment took place in health care facilities, whereas our study utilized web-based recruitment. Participants are likely to be more committed to the studies when recruited from their care facility and with the knowledge of their care physician [43]. In contrast, studies such as ours that recruited participants through the web may experience a quick loss to follow-up due to a less structured environment [6,11]. Future studies with My Care Hub might consider recruitment from a structured setting as a further strategy to improve participants’ retention.

Retention was not influenced by participant characteristics measured, with the exception that unemployed participants were less likely to complete the study, which was contrary to the results of a previous mHealth study [44]. Reasons for this discrepancy are unclear, although despite this difference, 50% of unemployed participants were retained in this study, which is relatively high for web-based interventions. Future research with My Care Hub will explore reasons for higher attrition among unemployed participants and the use of empirical strategies to improve their retention rates.

### Intervention Engagement

Users in our study actively used the app for 11 of 14 days (11/14, 79%) in the first 2 weeks, where they all used the app at least once during the first week and 85% returned to use the app during week 2 and 71% during week 3. To put these rates into perspectives, we refer to studies of Faridi et al [45] and Kim et al [42], who found that 53% and 38%, respectively, of participants used the app for a portion of the 12 weeks intervention duration, where in some cases, there was up to 33% of completely inactive participants [45]. In comparison, our app frequency usage rate can be interpreted as reasonable. However, mobile-based interventions differ widely in terms of population, features, settings, and techniques used to foster engagement. For example, although our intervention was self-directed, and we did not utilize reminders for self-management or data entry, the above-mentioned studies used face-to-face intervention orientation [42,45], automated reminders for diabetes management [45], and physician review of adherence [42]. These disparities may have been a major influence on usage, making direct comparison with other app-based interventions difficult. However, the sharp reduction in app usage during week 3, where only 71% retuned to use the app without the push notifications reveals the role of push notification as a feature that could further stimulate users’ engagement with apps [46], especially those with content containing insights into how to overcome barriers to achieving health goals [47] as provided in this study. Nevertheless, some users find push notifications intrusive and annoying, especially when too frequent, thus limiting engagement with the intervention [48]. Hence, health apps should be built in ways that patients can customize and review when they see notifications or adjust the timing to suit the selected period of specific self-management tasks such as physical exercise or BG monitoring.

The intensity of usage showed that participants interacted more with features for monitoring of BGL and physical activities, which are in congruence with previous studies [5,49]. This was confirmed in the interviews where participants mentioned that these documentation features improved accountability for their self-management activities. This may be due to patients’ understanding of the importance of these self-management activities for optimal health outcomes. Another explanation might be because the documentation features were accompanied by analytics that foster improved awareness of BGLs, accountability, and better clarity of self-management activities, as mentioned in the interview. These behavioral strategies in the documentation and analytic features might have encouraged personal reflection among participants, hence the increased intensity of usage.

The active time spent on the documentation features demonstrated that the duration of app usage necessary to generate consistency is a parameter that depends on individual users [50]. This was reflected in the interviews where the pattern of use was denoted by users’ decision on sequence and DSM routine. This result reveals the advantage of a multicomponent intervention such as My Care Hub, which offers users the opportunity to embrace it in ways most relevant to their needs [51]. A user can bypass a feature that they feel does not apply to them, potentially increasing engagement with more relevant areas in relation to their needs. Therefore, the diverse elements available in My Care Hub represent an advancement over many existing diabetes app interventions that consist of only a single element that requires participants to complete a predefined behavioral program [52].

Although the My Care Hub system log recorded participants’ passive usage of the education textual screens, there are no standard measures to compare these data with similar diabetes-focused interventions. However, the interviews indicated that participants appreciated this feature as an important element that provided knowledge reinforcement as a behavioral strategy for DSM. Nonetheless, the app system was unable to capture whether participants were actually reading and comprehending the embedded information or simply clicking them. An approach to address this limitation is to incorporate eye-tracking technology [53] or tailored quizzes [54] into My Care Hub to measure cognitive responses and knowledge acquired through engagement with each information screen. These measures would need to determine if success or failure of a user to acquire knowledge is due to the intervention component delivery mode, users’ engagement with the information, or some other intrinsic factors exclusive to the user.

Generally, engagement indices were initially high but decreased in subsequent weeks. Previous studies using mHealth interventions over short- and long-term periods have identified similar trends [52,55]. This finding was expected, as this study was a real-life pragmatic pilot testing of an app, prone to nonuse or infrequent use because users prefer to engage with apps periodically [55]. In addition, nonusage attrition with mHealth could be due to other reasons such as lack of self-motivation or commitment to change health behaviors [55] and satisfactory attainment of knowledge or skills in managing the disease [52].

Participants’ perceptions related to behavioral change strategies in My Care Hub derived from the documentation, feedback response, calories in foods, and education tips features are consistent with the needs analysis study conducted as part of the predevelopment phase of the app [25]. Both type 1 and type 2 diabetes patients expressed a strong interest in these elements because of their ability to not only foster engagement with an app but also provide benefits for self-management behaviors. This reinforces the notion that benefits derived from an intervention strongly affect users’ experience and, hence, engagement with the technology [23]. As these elements are targeted toward self-monitoring of behavioral activities and the provision of educational information to support those activities, the perceived behavioral change strategies may be an indicator that the app has the tendency to support users to achieve their behavioral goals. Nevertheless, further long-term studies are required to establish this claim.

Perceived ease of use of mHealth positively affects continuance in intention to use [56]. The presentation and characteristics of a technology determine the way users can optimize the elements to achieve their aim and overall behavioral goal [24]. If users enjoy their experience in a digital behavioral intervention, exposure to the behavioral change component will be improved and may subsequently influence behaviors [22]. These were reflected in our study as participants expressed their opinion about the simplicity of My Care Hub and perceived it as uncomplicated and effortless to use. Even when engagement is a purposeful choice and evolves from how people choose to obtain value from their experience, it has to be enabled by the technology and, thus, impacts long-term interaction with such technology [14].

The educational component of the app was informed by our previous study, which shows that information on basic guidelines for the management of diabetes and approaches to problem solving in diabetes were highly desired by both type 1 and type 2 diabetes patients [25]. However, once that knowledge is obtained, there is a tendency for a drop in participants’ rate of use of the app [57]. This highlights that apart from developing an app to meet end-user requirements and perceived relevance to diabetes management, mHealth developers need to consider ongoing novel strategies that will keep participants engaged. Novelty is also a main contributor to app engagement because it prevents boredom [23,58]. The downward trend in engagement indices may be explained by a lack of novelty in the app throughout the study period. Hence, future long-term research with My Care Hub must consider ongoing novel strategies that will keep participants engaged. Such strategies may be achieved by considering the suggestions raised by participants in this study. These include periodic information updates on meals and their corresponding nutrient values. Other suggestions on extended functionality in accessing more historical data, automated data transmission, and feedback on physical activity performance are also potential future improvements of My Care Hub, as they have been proven to have an effect on behavior [58].

### Strengths and Limitations

A mixed methods study design was used to evaluate patient engagement with My Care Hub, which is a strength of the study compared with previous studies that have arbitrarily classified engagement as high or low based on frequency of use [52] or overall adherence to the intervention [59]. The unique contribution of this paper is threefold. First, retention with My Care Hub indicates its potential as a relevant behavior change intervention tool for patients with diabetes in rural or remote environments with poorer access to specialist health care services. Second, participants’ engagement based on interaction with multiple intervention elements was measured using the FITT metrics. The use of this measure reveals the level of user engagement with each intervention feature, thus providing results that are beneficial to inform future enhancements of My Care Hub. Although FITT is commonly used in physical activity research [33], to the best of our knowledge, this is the first study to use this measure to assess users’ engagement with a multi-component DSM app. Adjusting the index to measure engagement with the intervention in this study was possible because behavior metrics and physical activities were measured. The use of FITT as a measure provided results that could broadly serve as a reference to evaluate other diabetes mHealth interventions before the execution of a full-scale trial. Third, due to the short intervention period of this study, we employed a theoretical and conceptual framework to confirm the components of BIT present in My Care Hub, as an analog to measures of behavioral engagement with the app. Therefore, the framework served as a predictive device to evaluate the app’s suitability as a behavior change intervention tool. This approach supports a more comprehensive assessment of engagement than most existing short-term pilot studies, which lack theoretical foundations. The use of this framework provides guidance on aspects of mHealth interventions to ensure the development of a meaningful tool that could improve patient engagement with healthy behaviors [24].

This study has some limitations that should be taken into account when interpreting the findings. The short intervention period is acknowledged. However, 3 weeks is the minimum time required for anyone to form a behavioral habit [60], and multiple components as found in our intervention are potentially effective techniques to achieve behavior change [61]. Furthermore, participant recruitment was restricted to a single source, and the sample size was small, thus limiting the sample diversity and generalizability of the results. In addition, the requirement that eligibility includes access to both an Android smartphone and an active email account may imply that the findings may not be generalizable to all smartphone users. In addition, because of the need for our app to comply with the Australian privacy policy and best practice on users’ confidentiality [62], we were unable to include programming codes within the app that could capture users’ personal profiles such as age, gender, browser, connection speed, etc. Having this information could provide an opportunity to assess different levels of engagement between those who completed the study and those who did not. In addition, we would have been able to assess if app use was moderated by users’ profile. Despite these limitations, considering the promising results further research with a larger sample and over an extended period of time is necessary.

### Conclusions

This study provided a comprehensive understanding of participant retention, technology usage, behavioral change process, and engagement with My Care Hub app during a short trial period. Retention was high, although further strategies may be required to further sustain retention when the app is used in long-term trials. The system log indices of FITT of engagement reveal a reasonable level of technology usage during the intervention period. The BIT model employed to measure behavioral change and engagement suggests that My Care Hub could be a behavior change intervention tool to support self-management behaviors in people with type 1 or type 2 diabetes. Information obtained through the use of multicomponent measures of engagement in this study provides rich and useful data regarding the strengths and weaknesses of My Care Hub and areas requiring improvement to foster increased engagement, sustainable long-term use, and effective health behavioral intervention.

## Appendix

Multimedia Appendix 1 Sample screenshots of the My Care Hub app.

Multimedia Appendix 2 Semistructured interview guide.

Multimedia Appendix 3 Consolidated criteria for reporting qualitative research checklist.

## Abbreviations

BG: blood glucose
BGL: blood glucose level
BIT: behavioral intervention technology
DSM: diabetes self-management
e-gift: electronic gift
FITT: frequency, intensity, time, and type
mHealth: mobile health
