# Supplementary figures and images for: User Retention and Engagement With a Mobile App Intervention to Support Self-Management in Australians With Type 1 or Type 2 Diabetes (My Care Hub): Mixed Methods Study
Source: JMIR Mhealth Uhealth. 2020 Jun 11;8(6):e17802. doi: 10.2196/17802 (PMC7317626; doi:10.2196/17802)

## Appendix 1: My Care Hub Screen Shots

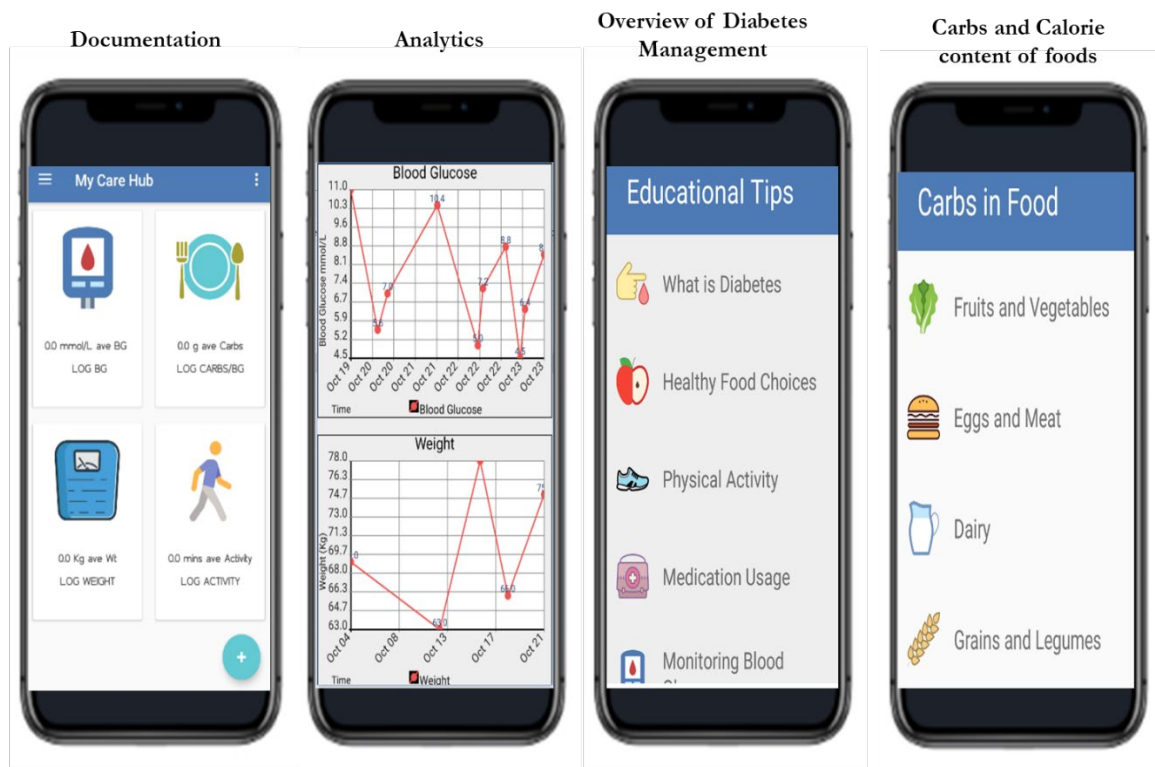

## Sample Feedback Messages

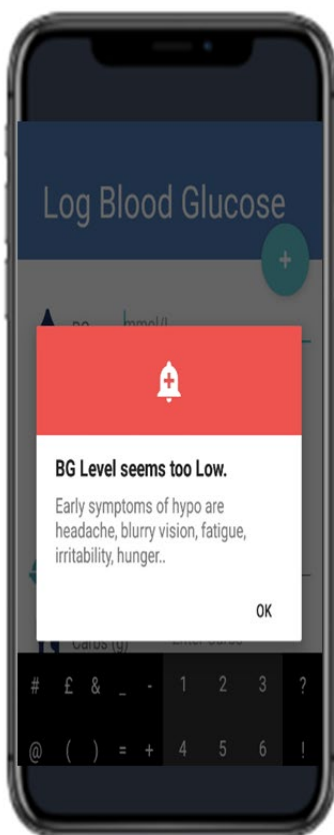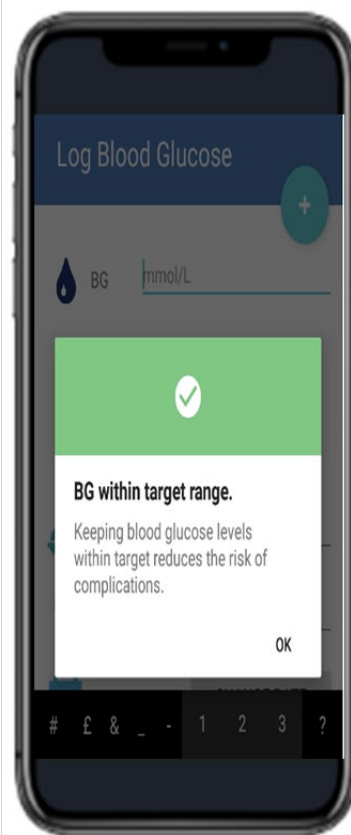

## Sample Push Notification

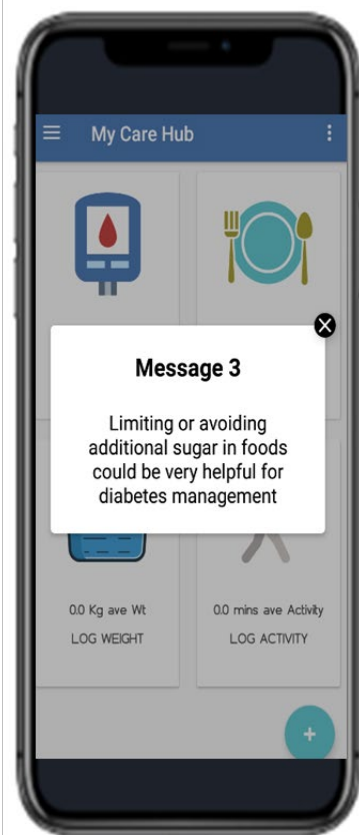

Supplement: Multimedia Appendix 1 [file mhealth_v8i6e17802_app1.pdf]
