# Supplementary material for: User Retention and Engagement With a Mobile App Intervention to Support Self-Management in Australians With Type 1 or Type 2 Diabetes (My Care Hub): Mixed Methods Study
Source: JMIR Mhealth Uhealth. 2020 Jun 11;8(6):e17802. doi: 10.2196/17802 (PMC7317626; doi:10.2196/17802)
Supplement: Multimedia Appendix 2 [file mhealth_v8i6e17802_app2.pdf]

Interview Guide

Prologue

1. Introduce myself and the reason for calling e.t.c

Set the focus of the interview and explain the app and the previous 3 weeks' study. Use the following script:

''Thank you for agreeing to take part in this interview and for using My Care Hub app in the past 3 weeks. I want to understand what you think about the app. The app is aimed to provide support and education to foster diabetes self-management by providing a platform to document and monitor self-management activities, give feedback on inputted blood glucose levels per time, provide information within the app on how to self-manage diabetes. In this interview, we aim to know your opinion and experience with the app during the course of usage. There are no right or wrong answers to my questions.''

2. Give opportunity for questions (if any),

3. Explain that the interview will be recorded:

''I would like to record what you say as that saves me having to scribble when you're talking and give opportunity for me to concentrate on what you're saying. The interview will be transcribed and your identity will be anonymised in the published work. Is that okay with you?

Questions.

Before you go into details about your experience with the app, I want to ask you some background information about your health and personal self-management

<u>Opening questions</u>

- Can you tell me, what type of diabetes you have and how long you have been diagnosed?
- Can you tell me how or the various steps you take to manage your diabetes? *e.g Therapeutic regimen, mediation intake, frequency of exercise, diet management?*

(*Please note, if participant has mentioned the type of diabetes from interview start, please refer to it in particular when asking these questions, instead of using the Phrase Your type of diabetes''*

<u>Experience of using My Care Hub</u>

- Did you use My Care Hub?
  - How often
  - If the app was not used, why?
- Describe your experience of learning how to use the app
  - Did you have any problem installing/using the app?
- Tell me about your experience with using My Care Hub app over the past 3 weeks?
  - How did you find navigating the pages and finding information?
  - How did you find inputting your data into the app?
  - Was there anything you find particularly hard to use?
  - If you have problems with the app, what were they?

- How did you find using My Care Hub for your diabetes self-management?

Views about features provided

- What are your views about the feature where you input your blood glucose?
  -How did you find inputting blood glucose into that feature?

- What are you views about the automated feedback messages received immediately after inputting your blood glucose into the app?
  -Were they useful?
  -What was your response to it?

- Did you use the analytic feature to monitor trends?
  -If not, why?
  -If yes, what are your views about it?
  - Did it help you in your diabetes management? How?

- What are you views about the diabetes educational tips embedded in the app? i.e the feature containing information on what diabetes is, carbohydrates in foods, healthy eating, exercise, medication usage, monitoring of blood glucose e.t.c
  -What do you think of them?
  -Did you find them useful?
  - Is there any other useful information that you would have liked to be included in the app?

- What are your views about the daily educational messages that are intermittently displayed in the app (push notifications)?
  -Was there anything you dislike about this messages in particular?
  - Did you find them useful?

- What feature(s) in the app did you find most useful? Why?

- What feature(s) in the app did you find least useful? Why?

- What do you think about the overall content and functionality of the App?

- Was there anything you disliked in the app?

Perceived ongoing benefit /Impact/intention for continue usage

- What ongoing benefit do you think there might be for patients with your type of diabetes who continue to use My Care Hub app?
- Is there anything you would do differently regarding your self-management following your participation in the study?
- What factors/features in the app interest you most which could help you engage regularly with the app.
- Were there benefits you derived from using the app during the intervention period?
- What are your views about the educational component of the app?
- Would you continue to use My Care Hub, if it were available?
- Would you recommend the app to other people with your type of diabetes?

<u>Recommendations</u>

- Do you have suggestions on how the app could be improved?
- Do you think any features should be removed from the app?

  -If yes, please explain
- Was there anything you wanted or expected to see in the app but didn't?
- What would you ask the app developers to change? E.g colour, layout, icons, font size, any feature.

Is there anything we haven't talked about that you think it is important to share before we end the interview?

Thank you for the valuable information and your time.
