# Supplementary material for: User Retention and Engagement With a Mobile App Intervention to Support Self-Management in Australians With Type 1 or Type 2 Diabetes (My Care Hub): Mixed Methods Study
Source: JMIR Mhealth Uhealth. 2020 Jun 11;8(6):e17802. doi: 10.2196/17802 (PMC7317626; doi:10.2196/17802)
Supplement: Multimedia Appendix 3 [file mhealth_v8i6e17802_app3.pdf]

Appendix 3. Consolidated criteria for reporting qualitative research (COREQ): 32-item checklist.

No. Item	Guide questions/description	Where in manuscript
**Domain 1: Research team and reflexivity**		
Personal Characteristics		
1. Interviewer/facilitator	Which author/s conducted the interview or focus group?	AD
2. Credentials	What were the researcher's credentials? E.g. PhD, MD	MDA: *BSc, Msc, Grad Cert Diab Edu*;<br>AD: Bsc, PhD<br>UHM: MBBS, *Msc, MD*;<br>AEOMA: *BSc, Msc, PhD;*<br>BMA: *BSc, Msc, Grad Cert ULT, Grad Cert Mgt, PhD*
3. Occupation	What was their occupation at the time of the study?	Please find at the end of this list
4. Gender	Was the researcher male or female?	MDA: Female<br>AD: Male<br>UHM: Male<br>AEOMA: Male<br>BMA: Female
5. Experience and training	What experience or training did the researcher have?	All authors were experienced researchers in qualitative studies and have taken part and published peer reviewed articles in mHealth for diabetes management.
Relationship with participants		
6. Relationship established	Was a relationship established prior to study commencement?	Methods
7. Participant knowledge of the interviewer	What did the participants know about the researcher? E.g., personal goals, reasons for doing the research	Methods

8. Interviewer characteristics	What characteristics were reported about the interviewer/facilitator? E.g., Bias, assumptions, reasons and interests in the research topic	The motivation and background of the study were made clear to the participants before the start of the interview. Participants had no prior relationship with the interviewer.
**Domain 2: study design**		
Theoretical framework		
9. Methodological orientation and Theory	What methodological orientation was stated to underpin the study? e.g. grounded theory, discourse analysis, ethnography, phenomenology, content analysis	Methods
Participant selection		
10. Sampling	How were participants selected? e.g. purposive, convenience, consecutive, snowball	Methods
11. Method of approach	How were participants approached? e.g. face-to-face, telephone, mail, email	Methods
12. Sample size	How many participants were in the study?	Results
13. Non-participation	How many people refused to participate or dropped out? Reasons?	Results
Setting		
14. Setting of data collection	Where was the data collected? e.g. home, clinic, workplace	Methods
15. Presence of non-participants	Was anyone else present besides the participants and researchers?	Methods
16. Description of sample	What are the important characteristics of the sample? e.g. demographic data, date	Results
Data collection		
17. Interview guide	Were questions, prompts, guides provided by the authors? Was it pilot tested?	Methods, Appendix 2
18. Repeat interviews	Were repeat interviews carried out? If yes, how many?	Methods
19. Audio/visual recording	Did the research use audio or visual recording to collect the data?	Methods
20. Field notes	Were field notes made during and/or after the interview or focus group?	None

21. Duration	What was the duration of the inter views or focus group?	Methods
22. Data saturation	Was data saturation discussed?	Methods
23. Transcripts returned	Were transcripts returned to participants for comment and/or correction?	Methods
**Domain 3: analysis and findings**		
Data analysis		
24. Number of data coders	How many data coders coded the data?	Methods
25. Description of the coding tree	Did authors provide a description of the coding tree?	Methods
26. Derivation of themes	Were themes identified in advance or derived from the data?	Methods
27. Software	What software, if applicable, was used to manage the data?	Methods
28. Participant checking	Did participants provide feedback on the findings?	Methods
Reporting		
29. Quotations presented	Were participant quotations presented to illustrate the themes/findings? Was each quotation identified? e.g. participant number	Results
30. Data and findings consistent	Was there consistency between the data presented and the findings?	Yes, Results
31. Clarity of major themes	Were major themes clearly presented in the findings?	Yes, Results
32. Clarity of minor themes	Is there a description of diverse cases or discussion of minor themes?	Yes, Results

Occupation of interviewer and researchers at the time of the study:

- Mary D. Adu: PhD Candidate, College of Medicine and Dentistry, James Cook University. Australia
- Aaron Drovandi: Lecturer, College of Medicine and Dentistry, James Cook University. Australia
- Usman H. Malabu: Consultant Endocrinologist and Professor of Medicine, Townsville Hospital and Health Services / College of Medicine and Dentistry, James Cook University. Australia
- Aduli EO. Malau-Aduli: Associate Professor, College of Public Health, Medical and Veterinary Sciences, James Cook University.
- Bunmi S. Malau-Aduli: Associate Professor, College of Medicine and Dentistry, James Cook University. Australia.

Reference: Tong A, Sainsbury P, Craig J. Consolidated criteria for reporting qualitative research (COREQ): a 32-item checklist for interviews and focus groups. *International Journal for Quality in Health Care*. 2007. Volume 19, Number 6: pp. 349 – 357
